# Integrated Genetic Mapping, Transcriptomic, and Small RNA Analysis of Fruit Bitterness in Melon

**DOI:** 10.3390/ijms27209141

**Published:** 2026-10-15

**Authors:** Ligong Liu, Guoliang Yuan, Mengqing Lu, Weiqi Han, Haijun Zhang, Jianshe Wang, Huijun Zhang, Congcong Li, Jian Ma

**Affiliations:** 1 Key Laboratory of Vegetable Biobreeding, Beijing Vegetable Research Center, Beijing Academy of Agriculture and Forestry Sciences, Beijing 100097, China; 2 College of Horticulture Science and Technology, Hebei Normal University of Science and Technology, Qinhuangdao 066000, China; 3 School of Life Science, Huaibei Normal University, Huaibei 235000, China; 4 Biotechnology Research Institute, Chinese Academy of Agricultural Sciences, Beijing 100081, China

**Keywords:** melon, bitterness, cucurbitacin, *CmBr*, multi-omics

## Abstract

Melon fruit bitterness, mainly caused by cucurbitacin B (CuB), is a major quality defect that reduces consumer acceptance and market value. However, the genetic and regulatory mechanisms underlying fruit bitterness remain largely unclear. In this study, two thin-skinned melon inbred lines, A54 (bitter leaves and fruit) and B15 (bitter leaves but non-bitter fruit), were used to dissect the genetic basis of fruit bitterness. Genetic analysis revealed that fruit bitterness in A54 is controlled by a single dominant locus. Through bulked-segregant analysis sequencing (BSA-seq) and fine mapping, the candidate region was delimited to a 133-kb interval on chromosome 9, which contains four bHLH genes. Among them, *MELO3C005610* (formerly designated *CmBr*) exhibited 25.5-fold higher expression in A54 than in B15 and was identified as the most plausible positional candidate gene. We further developed a near-isogenic line with bitter fruit (B15^NIL^) and performed integrated transcriptome, small RNA, and degradome sequencing. Twelve CuB biosynthetic genes were significantly up-regulated in B15^NIL^ fruits, indicating coordinated activation of the cucurbitacin biosynthetic pathway. In addition, small RNA and degradome analyses identified candidate miRNA-target interactions that may contribute to post-transcriptional regulation of CuB biosynthesis, although these interactions require further functional validation. Together, these findings provide a framework for understanding the genetic basis of fruit bitterness in melon and suggest candidate targets for future functional validation and breeding of non-bitter cultivars.

## 1. Introduction

Melon (*Cucumis melo* L.) is an economically important cucurbit crop cultivated worldwide, valued for its sweet fruit, rich nutritional value, and unique aroma [1,2,3]. Melon has been classified into two subspecies: *C. melo* ssp. *melo* and *C. melo* ssp. *agrestis* based on ovary pubescence [4,5,6,7]. Typically, young fruits of most *ssp*. agrestis accessions are bitter, with bitterness progressively diminishing during fruit maturation [3]. Nevertheless, under production conditions, genetic, environmental, or cultivation-related factors can result in persistent fruit bitterness, thereby severely compromising melon fruit quality and reducing its commercial value [8]. Therefore, elucidating the genetic and molecular regulatory mechanisms underlying bitterness is of great significance for accelerating the breeding of non-bitter cultivars.

Bitter compounds are widely distributed in plants, and the bitterness in Cucurbitaceae plants is mainly caused by cucurbitacins [9]. Cucurbitacins are highly oxygenated tetracyclic triterpenoids that serve as defensive compounds against herbivores and plant pathogens and also possess pharmacological activities [10,11,12,13]. In melon, cucurbitacin B (CuB) is the predominant bitter compound, whereas cucurbitacin C (CuC) and cucurbitacin E (CuE) dominate in cucumber and watermelon, respectively [14,15,16,17,18,19]. Chemically, CuB (C32H46O8) possesses a cucurbitane-type skeleton, specifically 19-(10→9β)-abeo-10α-lanost-5-ene (also known as 9β-methyl-19-norlanosta-5-ene). It has nine stereocenters and one E/Z center (Δ23), with β-OH at C-2, α-OH at C-16, keto groups at C-3, C-11, and C-22, and double bonds at C-5/C-6 and C-23/C-24. Its acetylated C-25 hydroxy group distinguishes it from the non-acetylated precursor CuD.

The biosynthesis and regulation of cucurbitacins have been well studied in Cucurbitaceae crops such as cucumber and melon [18,19,20]. In cucumber, a nine-gene CuC biosynthetic module includes an oxidosqualene cyclase (OSC, *Bitterness*, *Bi*), seven cytochrome P450s (CYPs), and an acyltransferase (ACT), which are co-expressed and partially clustered on chromosome 6 [9,19]. In addition, two basic helix–loop–helix (bHLH) transcription factors, *Bitter leaf* (*Bl*) and *Bitter fruit* (*Bt*), regulate CuC biosynthesis in leaves and fruits, respectively, and mutations in the *Bt* promoter are responsible for the non-bitter phenotype of cultivated cucumber [9]. Comparative genomics revealed conserved syntenic loci for cucurbitacin biosynthesis in cucumber, melon, and watermelon and identified a syntenic bHLH gene cluster regulating tissue-specific cucurbitacin biosynthesis [19]. In melon, the CuB biosynthetic gene cluster is located on chromosome 11, containing an OSC (*Cucumis melo Bitterness*, *CmBi*), four CYPs, and an ACT (*CmACT*) gene, while the two bHLH genes, *Cucumis melo Bitter root* (*CmBr*) and *Cucumis melo Bitter fruit* (*CmBt*), are located on chromosome 9 and were initially proposed to regulate CuB biosynthesis in roots and fruits, respectively [17,18,19]. However, no sequence variations associated with their function have been identified in melon.

Recent studies have further refined the mechanisms by which *CmBt* and *CmBr* regulate CuB biosynthesis. Luo et al. showed that 20 mg/L CPPU promotes CuB accumulation by inducing *CmBt*, which in turn activates the expression of genes such as *CmBi*, *Cm710*, and *CmACT* [8]. However, Wang et al. demonstrated that *CmBr*, rather than *CmBt*, is the major regulator of CPPU-induced CuB biosynthesis in fruit [21]. Xu et al. also analyzed 1160 bHLH genes across seven cucurbit crops and found that the *Bt* cluster originated from three ancestral genes after cucurbit-common tetraploidization and identified a paralogous cluster containing *Brp*, which regulates cucurbitacin biosynthesis in roots [22]. Wang et al. used CRISPR/Cas9 to generate *Cmbt* knockout and demonstrated that *CmBt* predominantly regulates CuB accumulation in leaves, whereas *CmBr* plays a more prominent role in fruits and roots, redefining their tissue-specific roles [20]. They further showed that CmBt physically interacts with CmBr, and the CmBt-CmBr module synergistically activates *CmBi*.

Despite these advances, the molecular mechanism underlying melon fruit bitterness remains incompletely understood. Although Li et al. [23] previously identified a 62-kb presence–absence variation (PAV) on chromosome 9 that is absent in non-bitter melon and encompasses nine consecutive genes, including five cytochrome P450 genes, immediately upstream of *CmBr* and *CmBt*, the genetic basis of natural variation, the causal gene(s) at the fruit bitterness locus, and the miRNA-mediated regulatory network have not been systematically investigated. In this study, we used two thin-skinned melon inbred lines, A54 (bitter in both leaves and fruit) and B15 (bitter leaves but non-bitter fruit), to investigate the genetic inheritance of fruit bitterness. Through bulked-segregant analysis sequencing (BSA-seq) and gene fine mapping, we delimited the candidate region to a 133-kb interval on chromosome 9. Within this interval, *MELO3C005610* showed 25.5-fold higher expression in A54 than in B15 and was identified as the most plausible positional candidate gene. We also developed a near-isogenic line (B15^NIL^) and conducted integrated sequencing of transcriptome, small RNA, and degradome. Multiple CuB biosynthetic genes were found to be up-regulated in B15^NIL^ fruits, and we also identified differentially expressed miRNAs that potentially target these genes, which suggests a complex miRNA-mediated regulatory network. These findings provide a comprehensive framework for understanding the genetic and regulatory basis of fruit bitterness in melon and offer potential targets for breeding non-bitter melon cultivars.

## 2. Results

### 2.1. Inheritance of Fruit Bitterness in Melon

**Figure 1:**
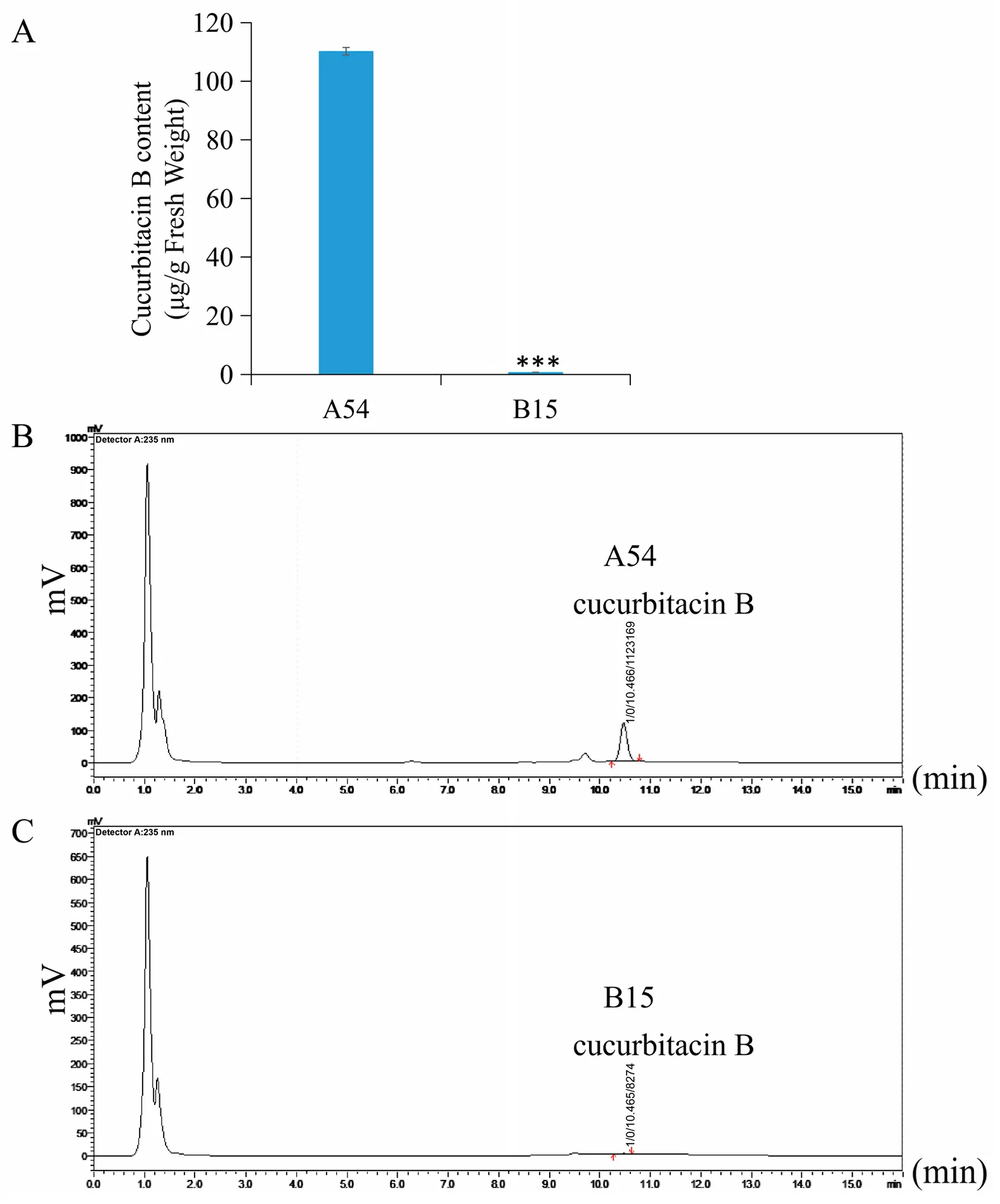
Cucurbitacin B (CuB) accumulation in melon fruits of A54 and B15. (**A**) CuB content in A54 and B15 fruits (3 DAP) determined by HPLC. The values are means ± SD, n = 3. *** represents *p* < 0.001 determined by Student’s *t*-test. (**B**,**C**) Representative HPLC chromatograms of CuB in extracts of A54 and B15 fruits, respectively. Detection was performed at 235 nm, and the peak corresponding to CuB is labeled with its retention time (10.5 min).

The contents of CuB in young fruits (three days after pollination, 3 DAP) of melon inbred lines A54 and B15 were determined by HPLC (Figure 1). The results revealed that A54 accumulated 110.254 ± 1.273 μg/g fresh weight (FW) of CuB, whereas B15 contained only 0.017 ± 0.005 μg/g FW. To elucidate the inheritance pattern of bitterness in fruit, reciprocal crosses were performed between inbred lines B15 and A54. A total of 20 F_1_ plants were obtained from these reciprocal crosses, and all of them produced bitter young fruits without segregation, indicating that bitterness is dominant over non-bitterness. Among the 2653 plants in the F_2_ population, 1995 plants exhibited bitter fruit and 658 displayed non-bitter fruit, fitting a 3:1 Mendelian ratio (χ_0.05_^2^ = 0.055, *p* = 0.81). These results demonstrate that fruit bitterness in A54 is controlled by a single dominant locus, consistent with a previous study [23].

### 2.2. Identification and Candidate Gene Analysis of the Fruit Bitterness Based on BSA-Seq

**Figure 2:**
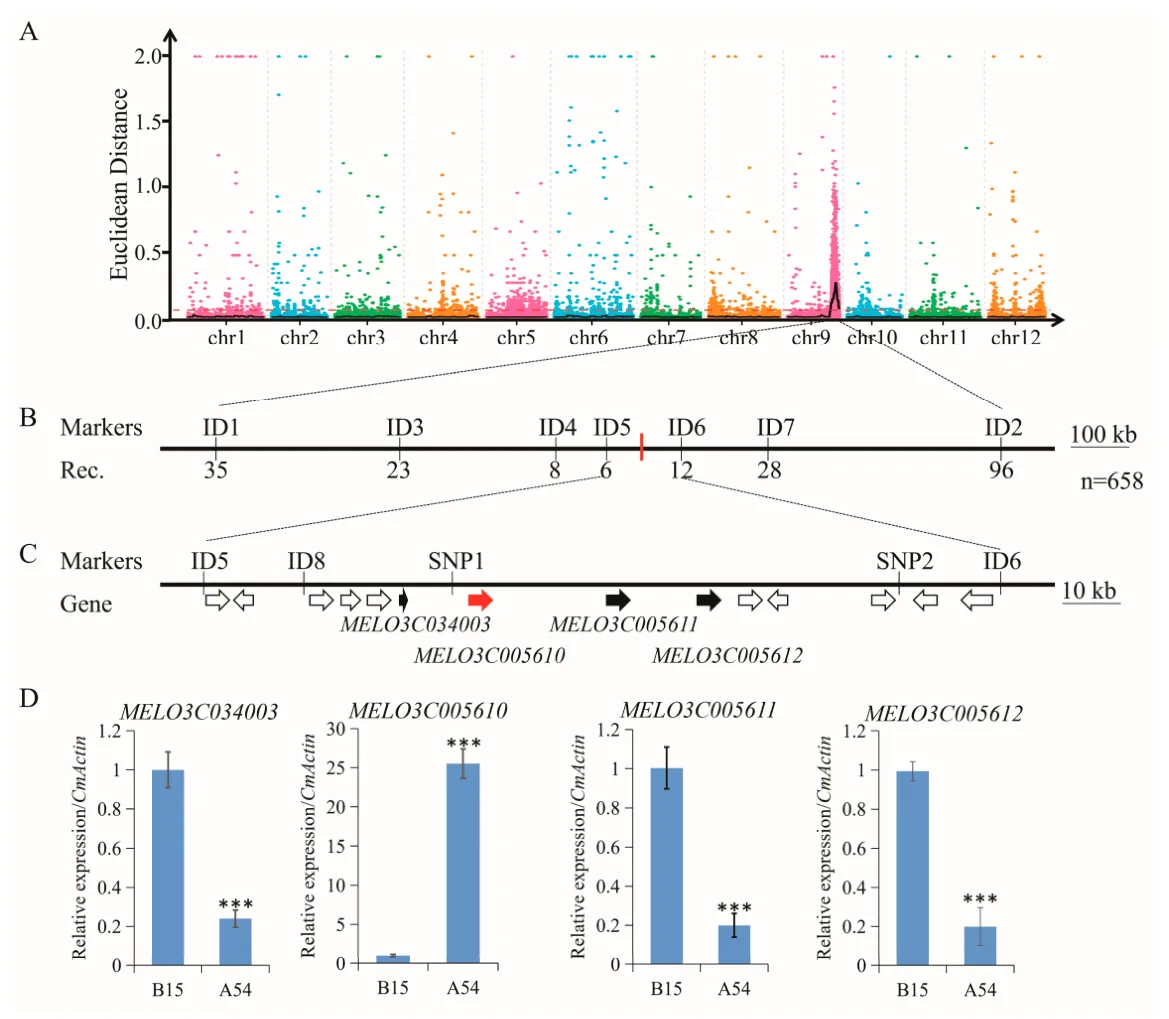
Genetic mapping and candidate gene analysis of fruit bitterness in melon. (**A**) Euclidean distance (ED) association plot from bulked segregant analysis sequencing (BSA-seq) conducted on the F_2_ population. The *x*-axis represents the 12 melon chromosomes, and the *y*-axis shows the corresponding ED values. (**B**,**C**) Fine mapping of the fruit bitterness locus was performed using molecular markers. The numbers below the markers indicate the number of recombinant individuals (Rec.). Arrows indicate the annotated genes within the interval; filled arrows represent the four bHLH transcription factor genes, and one red arrow highlights the potential candidate gene. (**D**) Relative expression levels of the four bHLH genes in 3 DAP young fruits of B15 and A54 determined by quantitative RT-PCR. The *CmActin* gene was used as the internal reference. *** represents *p* <  0.001 determined by Student’s *t*-test.

To quickly locate the locus of the target gene, the bulked-segregant analysis sequencing (BSA-seq) strategy was performed using 30 bitter and 30 non-bitter F_2_ individuals to construct the B-pool and NB-pool bulks, respectively. A total of 70,379,321 (20.51 Gb, 48×) and 74,205,154 (21.63 Gb, 51×) clean reads were obtained from the B-pool and NB-pool, respectively. Among them, 98.54% and 98.56% reads were mapped to the melon DHL92 (V4.0) reference genome. After filtering, 387,411 high-quality SNPs and 101,504 high-quality InDels were retained. Using the Euclidean distance (ED) algorithm, significant peaks were identified at 20.5–23.9 Mb for SNPs (ED value 0.15) and 20.8–23.5 Mb for InDels (ED value 0.24) on the terminal region of chromosome 9 (Figure 2A). The merged 2.7 Mb region (20.8–23.5 Mb) on chromosome 9 is likely the candidate region for the target gene controlling fruit bitterness in melon.

To confirm and narrow down the interval, eight InDel and two SNP polymorphic markers were developed from parental re-sequencing data. Subsequently, 658 non-bitter individuals were genotyped with these markers. Finally, the candidate region was delimited to a 133-kb interval (21,565,120–21,698,140 bp) flanked by markers ID5 and ID6, which co-segregated with ID8, SNP1, and SNP2 (Figure 2B,C). Fourteen putative genes were annotated in this region, including four bHLH transcription factor genes (*MELO3C034003*, *MELO3C005610*, *MELO3C005611*, and *MELO3C005612*) (Table 1, Figure 2C). *MELO3C034003* gene encodes an 84-amino-acid protein, whereas *MELO3C005610* and *MELO3C005611* were previously identified as *CmBr* and *CmBt* [19]. Subsequently, the coding regions of all fourteen putative genes were amplified and sequenced, but no sequence variation was detected between A54 and B15.

To further pinpoint the candidate gene, an approximately 89-kb genomic region between markers ID8 and SNP2 was analyzed based on the HS reference genome [24], because the DHL92 reference genome contained too many N nucleotides. Within this region, nine SNPs and three InDels were identified, all located in introns or intergenic regions. No variants were detected within the coding regions of the four bHLH genes, which is consistent with the Sanger sequencing. SNP1, the closest SNP to *MELO3C005610*, is positioned 2633 bp upstream of the ATG start codon (Figure S1). In addition, two putative transposon proteins were independently predicted in the upstream regions of *MELO3C005610* and *MELO3C005612*, respectively (Figure S1). Moreover, quantitative real-time PCR (qRT-PCR) revealed that the expression level of *MELO3C005610* in A54 was 25.5-fold higher than that in B15, while the remaining three bHLH genes were barely expressed in A54 (Figure 2D). These results suggest that *MELO3C005610* is the most plausible positional candidate gene underlying the fruit bitterness phenotype of A54.

### 2.3. RNA-Seq Analysis Between B15 and B15^NIL^

**Figure 3:**
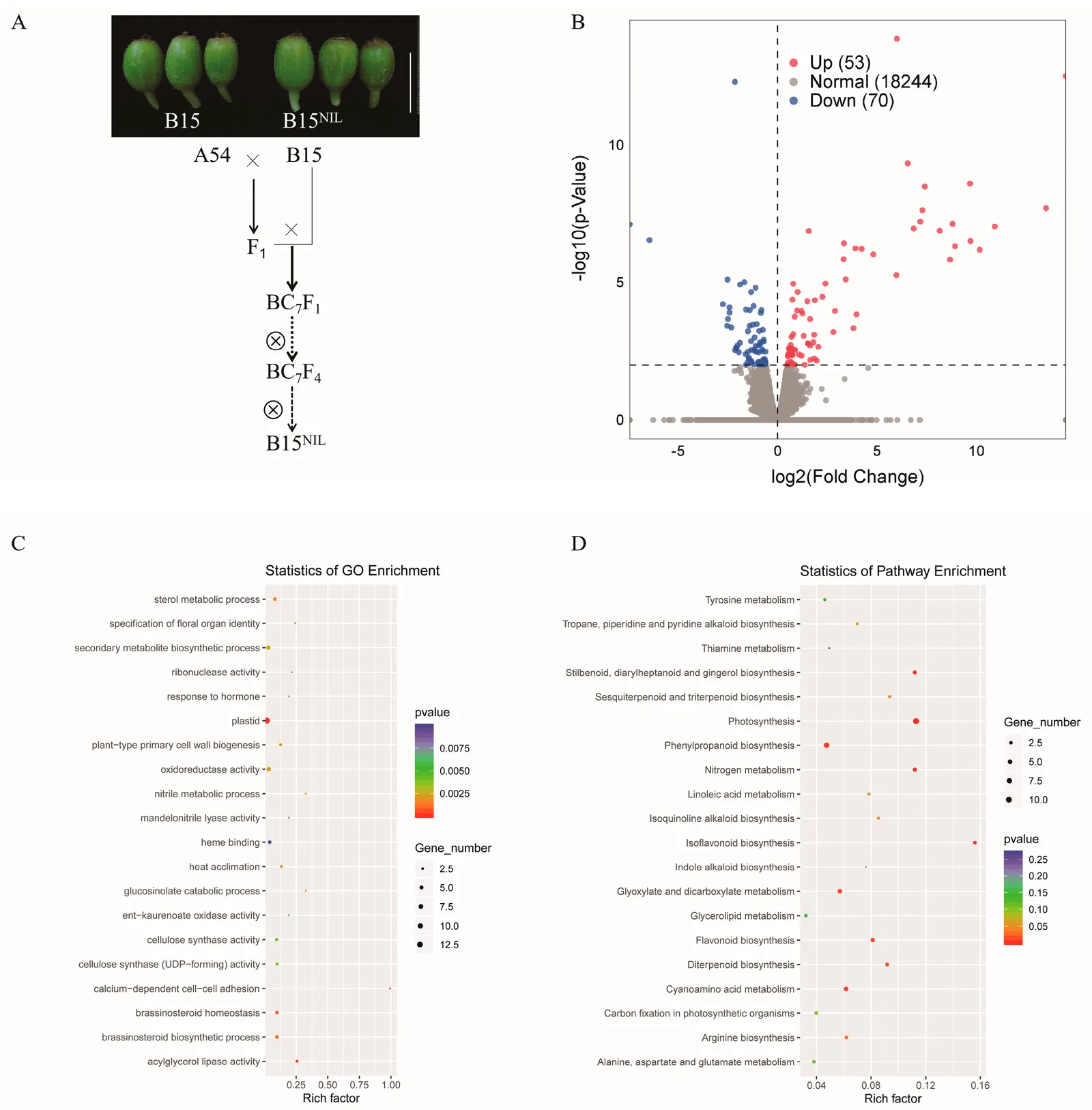
Transcriptome analysis of young fruits (3 DAP) from B15 and its near-isogenic line B15^NIL^. (**A**) Schematic representation of the near-isogenic line (NIL) construction and representative young fruits (3 DAP) of B15 and B15^NIL^. Scale bar = 2 cm. (**B**) Volcano plot of differentially expressed genes (DEGs) between B15 and B15^NIL^. (**C**) Gene Ontology (GO) enrichment analysis of DEGs. (**D**) Kyoto Encyclopedia of Genes and Genomes (KEGG) pathway enrichment analysis of DEGs.

To explore the regulatory network associated with fruit bitterness, a bitter fruit near-isogenic line, designated B15^NIL^, was developed from A54 in the B15 background (Figure 3A). Then, RNA sequencing (RNA-seq) was performed on young fruits (3 DAP) collected from B15^NIL^ and the recurrent parent B15 (Table S1). A total of 123 differentially expressed genes (DEGs), including 53 up-regulated genes and 70 down-regulated genes, were identified in B15^NIL^ compared with B15 (Table S2; Figure 3B). The GO enrichment analysis revealed that DEGs were particularly enriched in plastid development, oxidoreductase activity, and secondary metabolite biosynthetic process (Figure 3C). KEGG pathway enrichment analysis showed that DEGs were mainly enriched in photosynthesis, phenylpropanoid biosynthesis, glyoxylate and dicarboxylate metabolism, nitrogen metabolism, and stilbenoid, diarylheptanoid and gingerol biosynthesis (Figure 3D).

**Figure 4:**
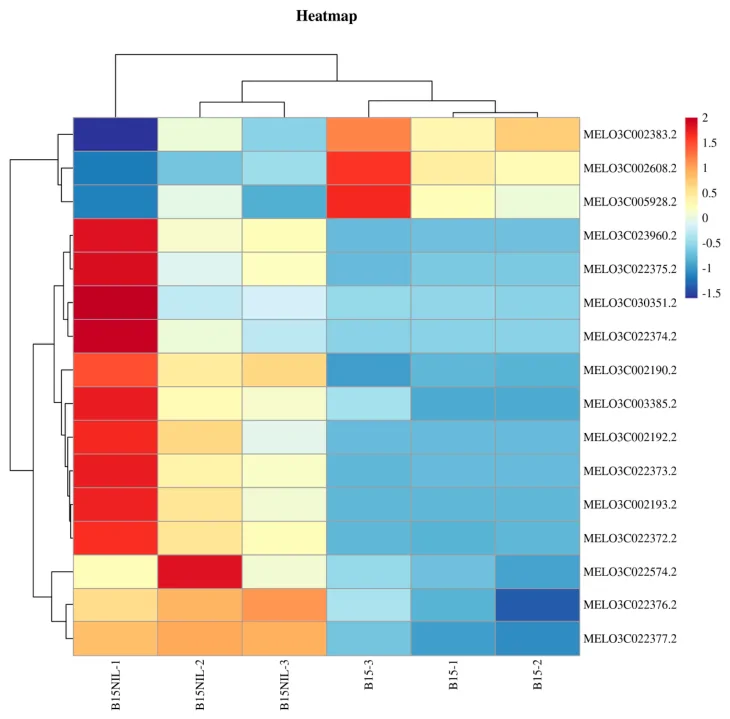
Heatmap of selected differentially expressed genes in 3 DAP young fruits of B15^NIL^ and B15. FPKM values were Z-score-normalized to visualize expression clustering. The *x*-axis represents samples (three biological replicates each), and the *y*-axis represents genes, including CuB biosynthetic genes and bHLH transcription factor genes. The Z-score scale ranges from −1.5 to +2.0, with colors changing from blue to red; blue indicates low expression and red indicates high expression.

Although no significant differential expression of the four genes (*MELO3C034003*, *MELO3C005610*, *MELO3C005611*, and *MELO3C005612*) was detected in the transcriptome data. 12 CuB biosynthesis genes were significantly up-regulated in the fruits of the B15^NIL^ compared with B15 (Table S3; Figure 4). These included the cucurbitadienol synthase gene *MELO3C022374* (*CmBi*), the multidrug and toxic compound extrusion gene *MELO3C002190* (*CmMATE1*) [25], two acyltransferase genes (*MELO3C002193* and *MELO3C022373; CmACT*), two beta-amyrin 11-oxidase-like genes (*MELO3C003385* and *MELO3C030351), and* six cytochrome P450 genes (*MELO3C002192*, *MELO3C022372*, *MELO3C022375*, *MELO3C022376*, *MELO3C022377*, and *MELO3C023960*). Furthermore, three other bHLH transcription factors (*MELO3C002383*, *MELO3C002608*, and *MELO3C005928*) were significantly down-regulated and another bHLH transcription factor *MELO3C022574* was significantly up-regulated in the B15^NIL^ (Table S4; Figure 4). These results suggest that these genes may also participate in the regulation of CuB biosynthesis in melon, although their specific roles require further functional validation.

### 2.4. Profiling of the Differentially Expressed miRNAs in Fruits of B15^NIL^ and B15

**Figure 5:**
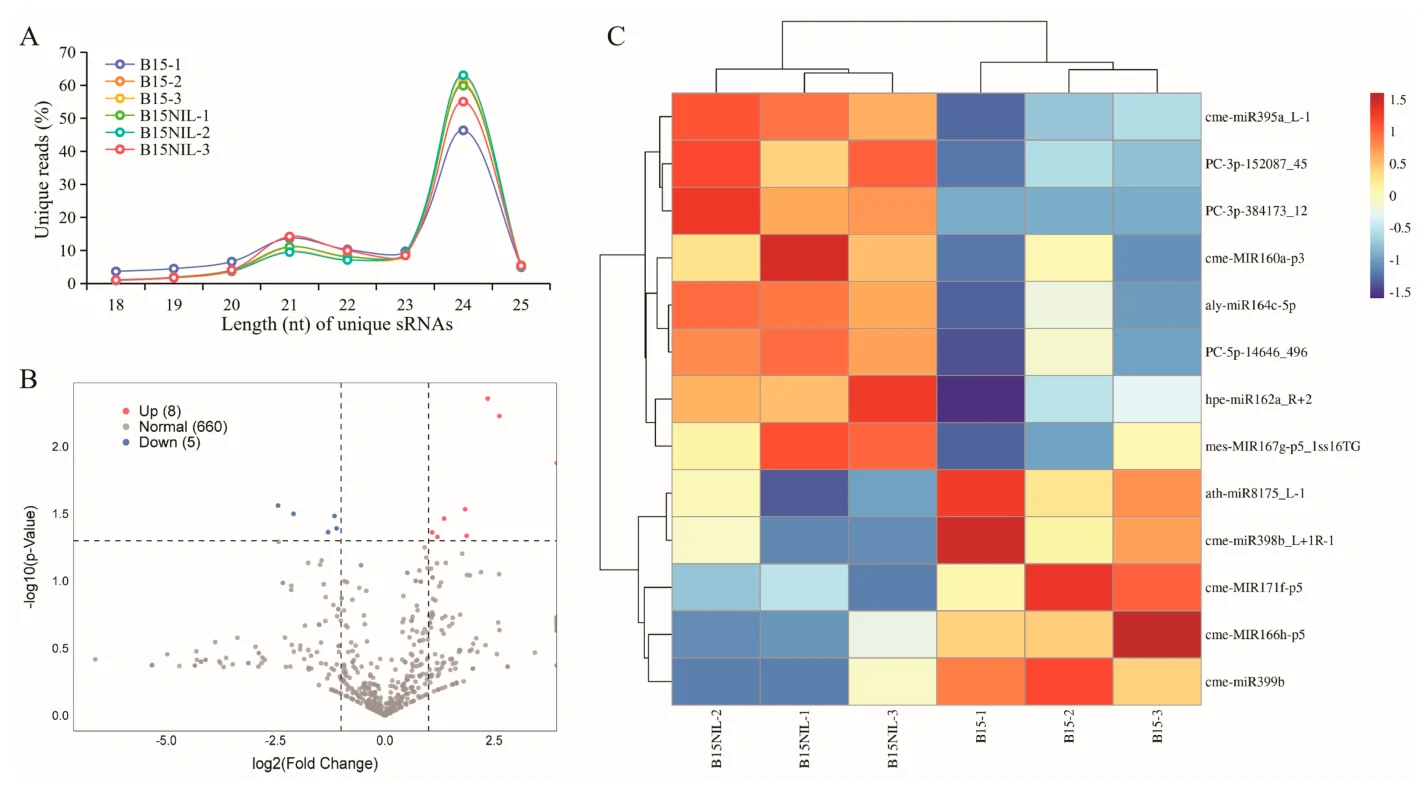
Small RNA sequencing analysis of young fruits (3 DAP) from B15^NIL^ and B15. (**A**) Length distribution of unique sRNAs from six libraries of B15 and B15^NIL^. (**B**) Volcano plot of differentially expressed miRNAs between B15^NIL^ and B15. (**C**) Heatmap of the differentially expressed miRNAs between B15^NIL^ and B15. The color scale represents Z-scores of normalized miRNA expression, ranging from −1.5 (blue, low expression) to +1.5 (red, high expression). The *x*-axis represents samples and the *y*-axis represents the differentially expressed miRNAs.

miRNAs participate in diverse plant developmental processes by regulating the abundance of their targets at the transcriptional or post-transcriptional level [26,27,28]. To identify miRNAs involved in regulating bitterness in melon, high-throughput small RNA sequencing was performed on six libraries with three biological replicates constructed from young fruits of B15^NIL^ and B15 (Table S5). Following the removal of low-quality reads and adapter sequences, clean reads were aligned against the Rfam database 14.0 to filter out rRNAs, snoRNAs, snRNAs, and tRNAs, yielding reads ranging from 18 to 25 nucleotides (nt) in length. The length distributions of unique sRNAs were highly similar across all six libraries, with 24-nt sRNAs being the most abundant, followed by 21-nt sRNAs (Figure 5A). Based on searches against the miRBase 20.0, a total of 358 known miRNAs and 315 novel miRNAs were identified, of which 110 were known miRNAs in melon (Table S6). These miRNAs were classified into 42 already documented miRNA families, with miR166 being the largest family comprising 23 members, followed by miR159 and miR156 comprising 21 and 19 members, respectively (Table S7).

Moreover, a total of 13 miRNAs exhibited differential expression patterns between B15^NIL^ and B15, of which eight were significantly up-regulated and five were significantly down-regulated (Table S8; Figure 5B,C). Of these, three up-regulated miRNAs, PC-3p-152087_45, PC-3p-384173_12, and PC-5p-14646_496, were annotated as novel miRNAs, whereas the remaining ten were known miRNAs. Additionally, cme-MIR166h-p5 was highly abundant, while cme-miR395a_L-1 and PC-3p-384173_12 were expressed at low levels, with the remaining miRNAs showing moderate expression levels. These results indicate that changes in CuB content are accompanied by alterations in miRNA expression profiles.

### 2.5. Identification of miRNA Target Genes via Degradome Sequencing

To ascertain the target genes of miRNAs, degradome sequencing was employed to precisely map cleavage sites within mRNA transcripts. Two degradome libraries, DB and DNB, were constructed from 3 DAP young fruits of B15^NIL^ and B15, and subsequent sequencing generated 19.2 million and 14.7 million raw sequencing reads, respectively. Finally, a total of 15,431 targets corresponding to 6915 transcripts were identified for 651 miRNAs using the CleaveLand 4.0 pipeline [29]. Specifically, 5493 target genes corresponding to 631 miRNAs and 5169 target genes corresponding to 620 miRNAs were identified in the DB and DNB libraries, respectively (Table S9). Of the identified miRNAs, only 41 were found to target a single gene, while the remaining miRNAs targeted at least two targets. Additionally, nine known and 22 novel miRNAs, together with their targets, were exclusively detected in the DB library, whereas eight known and 12 novel miRNAs and their targets were exclusively detected in the DNB library. Further analysis revealed that cme-MIR164a-p3 and ath-MIR8183-p5_1ss4TA targeted 1867 and 805 genes, respectively, and thus were identified as the miRNAs possessing the most target genes in both DB and DNB libraries.

To identify miRNAs and their target genes associated with bitterness, we performed a correlation analysis between miRNA expression profiles and transcriptome data. The results showed that a total of 184 target genes were identified for five significantly down-regulated miRNAs (cme-miR399b, cme-MIR166h-p5, cme-MIR171f-p5, ath-miR8175_L-1, and cme-miR398b_L+1R-1) and four significantly up-regulated miRNAs (cme-miR395a_L-1, hpe-miR162a_R+2, mes-MIR167g-p5_1ss16TG, and cme-MIR160a-p3) (Table S10). However, the expression levels of these target genes showed no change between bitter and non-bitter materials. Subsequently, we further analyzed 12 genes involved in the CuB biosynthesis pathway and found that 17 miRNAs could target eight of these genes (Table S11). Among them, ath-MIR8183-p5_1ss4TA targeted *MELO3C003385*, *MELO3C022375*, and *MELO3C022376*, whereas cme-MIR164a-p3 targeted *MELO3C022375* and *MELO3C022376* (Table S11). The remaining miRNAs each targeted only one of these genes. In addition, *MELO3C002190* and *MELO3C023960* were each targeted by two miRNAs, and *MELO3C022373* and *MELO3C022375* were each targeted by three miRNAs, whereas *MELO3C022376* was targeted by seven miRNAs (Table S11, Supplementary data). These observations collectively imply that the cucurbitacin biosynthetic pathway is subject to complex miRNA-mediated regulation. Although the lack of changes in target gene expression suggests that miRNAs may not be the main driver of bitterness variation in this population, or that post-transcriptional regulation is masked by other regulatory layers, our findings nonetheless indicate that CuB biosynthesis may be regulated by a relatively complex mechanism.

## 3. Discussion

Bitterness in melon fruit is a quality trait largely determined by the accumulation of CuB. In this study, we integrated genetic mapping, transcriptome profiling, small RNA sequencing, and degradome analysis to dissect the regulatory architecture underlying fruit bitterness in the melon inbred lines A54 and B15. Our genetic analysis demonstrated that fruit bitterness in A54 is controlled by a single dominant locus, and based on its expression pattern, we identified the bHLH transcription factor *MELO3C005610*, also known as *CmBr*, as the most plausible positional candidate gene. Furthermore, we demonstrated that the CuB biosynthetic pathway is transcriptionally activated in a bitter near-isogenic line (B15^NIL^) and is subject to post-transcriptional regulation by miRNAs. These findings provide a multilayered view of bitterness regulation and suggest that both transcriptional activation and miRNA-mediated fine-tuning contribute to CuB accumulation.

Bitterness in melon fruit is dominant over non-bitterness, and its genetic basis is relatively complex and influenced by environmental and hormonal factors [18,23]. Early studies identified four genes controlling bitterness in melon from different melon resources, namely *Bi*, *Bif-1*, *Bif-2*, and *Bif-3* [30,31,32]. Shang et al. showed that the melon bitterness trait is controlled by two completely dominant genes in the studied population, and three associated loci were mapped to chromosomes 2 and 5 based on 2b-RAD sequencing [33]. Li et al. detected a QTL on chromosome 9 for young fruit bitterness in melon that explained 20% of the phenotypic variation and identified a 62-kb PAV in non-bitter materials encompassing nine genes, including five CYP genes, and adjacent to *CmBr* and *CmBt* [23]. The inheritance pattern observed in the present study is consistent with that reported by Li et al. [23], namely that young fruit bitterness in melon is controlled by a single dominant locus. Using a BSA-seq strategy in this study, we mapped the target gene to a 2.7 Mb interval on chromosome 9 and further narrowed it to a 133-kb region containing fourteen annotated genes (Figure 2). Among these, four genes encode bHLH transcription factors, a family that is frequently involved in the regulation of plant secondary metabolism [19,34]. Notably, *MELO3C005610* and *MELO3C005611* were previously identified as *CmBr* and *CmBt*, reinforcing the relevance of this region to bitterness regulation [19]. The absence of coding DNA sequence variation between A54 and B15 suggests that the functional divergence at *CmBr* and *CmBt* is likely due to regulatory rather than protein-coding changes. Consistent with this, the expression level of *CmBr* in A54 was 25.5-fold higher than that in B15, whereas the other three bHLH genes were barely expressed in A54. Moreover, a SNP located 2633 bp upstream of the *CmBr* start codon, together with two predicted transposon elements in the upstream regions of *CmBr* and *CmBt*, may affect promoter activity or chromatin context. Wang et al. also showed that knockout of *CmBt* mainly reduced CuB content in melon leaves, resulting in non-bitter leaves, whereas *CmBr* introgression lines (*Cmbr^IL^*) significantly reduced CuB content in fruits, resulting in a non-bitter fruit phenotype [20]. Together, these observations suggest that *CmBr* acts as a positive regulator of CuB biosynthesis and that its expression divergence may underlie the bitter versus non-bitter fruit phenotype.

Transcriptome analysis of B15^NIL^ and B15 further revealed the downstream effects of *CmBr*/*CmBt* activation. Twelve genes involved in CuB biosynthesis were significantly up-regulated in B15^NIL^, including *CmBi*, *CmMATE1*, *CmACT*, six cytochrome P450 genes, and several other biosynthetic genes (Table S3). This coordinated up-regulation indicates that the bitter phenotype is not simply caused by a single rate-limiting enzyme, but rather reflects the broad activation of the entire cucurbitacin biosynthetic pathway. Interestingly, the four bHLH genes located in the *CmBr*/*CmBt* region did not show significant differential expression in the RNA-seq data, despite the clear difference in *CmBr* expression between A54 and B15 detected by qRT-PCR. This inconsistency may arise from multiple factors. First, expression differences among these four genes may actually exist. However, since the reference genome contains an excessive number of N bases in this region, and these genes share high sequence similarity, some raw reads may have been filtered out, leading to the false observation of no differential expression. Second, *CmBr* may regulate CuB biosynthesis through mechanisms that are not captured at the transcript level. For example, *CmBr* may physically interact with *CmBt* or other bHLH proteins to determine fruit bitterness, rather than exerting its function by altering *CmBr* transcript abundance. In this case, even if *CmBr* activity differs between B15^NIL^ and B15, RNA-seq will not be able to detect any expression difference. Third, other downstream regulators may compensate for the change, or independently drive, the bitter phenotype in B15NIL. In our RNA-seq analysis, three other bHLH genes (*MELO3C002383*, *MELO3C002608*, and *MELO3C005928*) were significantly down-regulated in B15^NIL^, while MELO3C022574 was significantly up-regulated. Although the functions of these genes in CuB biosynthesis are not yet known, their differential expression implies that the bitterness regulatory network may be more complex than a single *CmBr*-centered module. Fourth, CuB accumulation and *CmBr* expression in melon fruit may be restricted to specific tissue regions, such as the placental region. Therefore, the whole-fruit RNA-seq analysis conducted in this study may have masked cell-type-specific differences in *CmBr* expression. Taken together, these considerations indicate that the absence of *CmBr* differential expression in B15^NIL^ does not exclude its involvement in fruit bitterness, but it does mean that *CmBr* should be regarded as a strong positional candidate gene rather than a demonstrated causal gene. However, definitive proof of causality will require functional validation, such as promoter activity assays, allele co-segregation analysis in larger populations, CRISPR/Cas9 knockout, or complementation experiments.

Small RNA sequencing results suggest that bitterness may also be associated with widespread changes in miRNA expression. Thirteen miRNAs were differentially expressed between bitter and non-bitter materials, with eight up-regulated and five down-regulated, including both known and novel miRNAs (Table S8). The high abundance of cme-MIR166h-p5, together with the low expression of cme-miR395a_L-1 and PC-3p-384173_12, indicates that these miRNAs may have different regulatory roles in bitter fruits. These observations support the hypothesis that changes in CuB content are accompanied by reprogramming of the miRNA regulatory network. Degradome sequencing also provided candidate degradome-supported interactions for miRNA-mediated cleavage of target transcripts. A total of 651 miRNAs were assigned to 6915 transcripts, and only 41 miRNAs targeted a single gene, whereas the majority targeted two or more genes, indicating that the miRNA regulatory network in melon fruit is highly pleiotropic. Although 184 target genes were identified for the differentially expressed miRNAs, their mRNA levels did not differ between bitter and non-bitter materials (Table S10). This uncoupling between miRNA expression and target mRNA abundance may arise because some miRNAs repress translation without inducing transcript degradation, or because miRNAs are not the main driver of bitterness variation in this population. The differentially expressed miRNAs did not overlap at the name level with the miRNAs predicted to target CuB biosynthetic genes (Tables S8 and S11), suggesting that miRNAs may fine-tune CuB biosynthesis rather than determine the bitter versus non-bitter phenotype.

Furthermore, we also found that 17 miRNAs target eight genes in the CuB biosynthetic pathway, providing evidence for direct post-transcriptional regulation of cucurbitacin biosynthesis (Table S11). Notably, *MELO3C022376* is targeted by seven miRNAs, *MELO3C022372* and *MELO3C022375* by three miRNAs each, and *MELO3C002190* and *MELO3C023960* by two miRNAs each. This uneven distribution suggests that specific CuB biosynthetic genes, particularly cytochrome P450s, are subject to intense miRNA-mediated regulation. Such dense targeting indicates that these CuB biosynthetic genes may be subject to complex post-transcriptional regulation. However, these target predictions should be interpreted with caution, and future functional validation will be required to determine which of these interactions are biologically relevant.

Taken together, this multi-omics study clarifies the genetic and regulatory basis of fruit bitterness in melon. We identified a single dominant locus and proposed *MELO3C005610* (*CmBr*) as the most likely positional candidate gene. Bitter fruits exhibited coordinated activation of CuB biosynthetic genes, and the small RNA and degradome data provide candidate post-transcriptional interactions. However, the lack of overlap between differentially expressed miRNAs and CuB-targeting miRNAs suggests that miRNAs may not be the main driver of bitterness variation in this population. This combined regulation may explain why bitterness follows simple Mendelian inheritance at the locus level but shows greater complexity at the network level. These findings improve our understanding of cucurbitacin biosynthesis and suggest candidate targets for breeding melon with controlled bitterness.

## 4. Materials and Methods

### 4.1. Plant Materials and Growth Conditions

Two thin-skinned melon inbred lines (*C. melo* ssp. *agrestis*), A54 (bitter leaves and fruit) and B15 (bitter leaves but non-bitter fruit), were used in this study, both of which were obtained from the Beijing Vegetable Research Center, Beijing Academy of Agriculture and Forestry Sciences. The F_1_ generation was obtained by crossing A54 with B15, and the F_1_ plants were then self-pollinated to generate the F_2_ population for genetic analysis. In addition, the F_1_ generation was backcrossed to B15 for seven generations and self-pollinated for four generations to construct a near-isogenic line for fruit bitterness, designated B15^NIL^. All plants were hand-pollinated on the day of anthesis to obtain fruits. All materials were planted in the greenhouse at the Sijiqing Experimental Station of the Beijing Vegetable Research Center, under natural light with routine irrigation and pest control.

### 4.2. Fruit Bitterness Evaluation and Quantification of CuB

To determine the fruit bitterness phenotype, sensory evaluation was conducted following the method described by Horie et al. [17]. At least three panelists were invited to taste 3 DAP fruits and categorize the taste as either “bitter” or “non-bitter”. Each panelist rinsed their mouth with water after tasting every fruit. If all three panelists classified a tested fruit from an individual plant as “non-bitter”, all fruits from this plant were categorized as “non-bitter”; otherwise, all fruits from the plant were categorized as “bitter”. The “bitter” and “non-bitter” phenotypes were defined based on these sensory evaluation results.

To quantify CuB content, the CuB content in young fruits (3 DAP) was determined by high-performance liquid chromatography (HPLC) as previously described [8]. Briefly, 0.5 g of frozen fruit powder was homogenized with 1.5 mL methanol, vortexed for 30 min, and ultrasonically extracted at 20 °C for 60 min. The extract was centrifuged at 10,000× *g* for 10 min at 4 °C, and the supernatant was filtered through a 0.22 μm membrane. HPLC analysis was performed on a Shimadzu Prominence LC-20AD (Shimadzu Corporation, Kyoto, Japan) equipped with a UV detector set at 235 nm. The mobile phase consisted of 70% methanol and 30% ultrapure water (*v*/*v*) at a flow rate of 0.5 mL/min, and the injection volume was 10 μL. CuB was identified and quantified by comparing retention time and peak area with an authentic CuB standard. Three fruits per sample were measured, and the data are presented as mean ± SD (Table S12). Student’s *t*-test was used to compare CuB content between A54 and B15 using GraphPad Prism 5.0.

### 4.3. BSA-Seq and Gene Mapping

Thirty bitter and 30 non-bitter F_2_ individuals were selected to construct the B-pool and NB-pool, respectively. Genomic DNA was extracted from young leaves using a modified CTAB method [35]. Libraries were constructed and sequenced on the Illumina HiSeq 2500 platform (Illumina, San Diego, CA, USA). Clean reads were aligned to the melon DHL92 (V4.0) reference genome using BWA (v0.7.12) [36], after which SNPs and InDels were called using GATK (v3.7) [37]. The Euclidean distance (ED) algorithm was applied to identify candidate regions associated with fruit bitterness [38]. Significant peaks were determined based on the ED values, and the merged region was considered the preliminary candidate interval. Parental re-sequencing data were analyzed using the Integrative Genomics Viewer software (IGV, v.2.14.0), and InDel and SNP markers were subsequently developed. Primer pairs were designed using Primer-BLAST (www.ncbi.nlm.nih.gov, accessed on 10 May 2020). The 658 non-bitter F_2_ individuals were genotyped with these markers to narrow down the candidate region.

### 4.4. Gene Amplification and Expression Analysis

Genomic DNA sequences of the candidate genes in the parental lines A54 and B15 were amplified using KOD FX DNA polymerase (TOYOBO, Osaka, Japan), and the PCR products were sent to Beijing Biomed Gene Technology Co., Ltd. (Beijing, China). for Sanger sequencing. The obtained raw sequences were assembled using DNAMAN software (v6) and then aligned against the reference genome DHL92 (v4.0). Total RNA was extracted from young fruits (3 DAP) of A54 and B15 using a Quick RNA Isolation Kit (Huayueyang Biotech, Beijing, China), and reverse-transcribed using EasyScript^®^ One-Step gDNA Removal and cDNA Synthesis SuperMix (TransGen Biotech, Beijing, China) according to the manufacturer’s instructions. qRT-PCR was performed on an ABI PRISM 7500 system (Applied Biosystems, Foster City, CA, USA) using PerfectStart^®^ Green qPCR SuperMix (TransGen Biotech, China). The *CmActin* gene was used as the internal reference [2]. Relative gene expression was calculated using the 2^−ΔΔCt^ method [39]. Three biological replicates were performed for each sample, and significant differences were determined by Student’s *t*-test. The primers are listed in Supplementary Table S13.

### 4.5. Transcriptome Analysis

Total RNA was extracted from young fruits (3 DAP) of B15^NIL^ and B15 using Trizol reagent (Invitrogen, Waltham, MA, USA) with three independent biological replicates. The RNA quantity and purity were analyzed using a Bioanalyzer 2100 and RNA 6000 Nano LabChip Kit (Agilent, Santa Clara, CA, USA) with RIN number > 7.0. High-quality RNAs were used for the construction of cDNA libraries (Thermo Fisher, Waltham, MA, USA). The final cDNA library with an average insert size of 300 ± 50 bp was sequenced on the Illumina Novaseq™ 6000 platform (LC-Bio Technologies (Hangzhou) Co., Ltd., Hangzhou, China). Expression levels of mRNAs were calculated by counting fragments per kilobase of transcript per million mapped fragments (FPKM). The differentially expressed genes (DEGs) were selected with *p*-value < 0.05 and |log_2_ (fold change)| ≥ 1 as the significance threshold [40]. GO functional classifications of all the DEGs were annotated using the gene-set enrichment analysis (GSEA) toolkit v1.1 (https://www.omicstudio.cn/tool, accessed on 15 April 2019). KEGG enrichment analyses were performed using clusterProfiler v3.9 [41].

### 4.6. Small RNA Sequencing

Total RNA extraction (three biological replicates) and library construction of 3 DAP fruits from B15^NIL^ and B15 were performed by LC-Bio Technologies (Hangzhou) Co., Ltd. (Hangzhou, China). Sequencing was performed on the Illumina HiSeq 2500 platform. The raw sequencing data were analyzed using ACGT101-miR software v4.2 (LC Sciences, Houston, TX, USA). Subsequently, the reads with a length of 18–25 nucleotides were aligned against the Rfam v14.0 and Repbase v24.03 (www.girinst.org/repbase, accessed on 10 June 2019) databases to remove rRNAs, snoRNAs, snRNAs, and tRNAs. Known and novel miRNAs were identified by BLAST searches against miRBase 20.0 [42]. Differentially expressed miRNAs were identified with the criteria of |log_2_ (fold change)| ≥ 1 and *p*-value < 0.05.

### 4.7. Degradome Library Construction and Sequencing

Two degradome libraries, DB (bitter fruit) and DNB (non-bitter fruit), were constructed from 3 DAP fruits of B15^NIL^ and B15, respectively. Briefly, Poly(A) RNA was specifically captured from total RNA (20 μg) using poly-T oligo-attached magnetic beads through two rounds of purification. The 5′ adapters were ligated to the 5′ end of the degraded mRNA fragments using RNA ligase, followed by reverse transcription with a 3′-adapter 6N random primer to generate cDNA flanked by adapters at both ends. Subsequently, size selection and purification were performed using AMPure XP beads, and the cDNA was amplified by PCR to construct a library with insert sizes of 200–400 bp.

For degradome sequencing, the two constructed libraries were sequenced on the Illumina HiSeq 2500 platform using 50 bp single-end sequencing (LC-Bio Technologies (Hangzhou) Co., Ltd., China). Raw sequencing data were analyzed using ACGT101-DEG software v4.0 (LC Sciences, USA). Cleavage sites were mapped using the CleaveLand pipeline 4.0 [29]. T-plots were used to visualize the target mRNAs of the identified miRNAs. Targets were classified into categories 0–4 based on the relative abundance of degradome reads at the cleavage site [43,44].

## Figures and Tables

**Table 1:** The annotated genes located in the candidate genomic regions associated with fruit bitterness.

Number	Gene Name	Function
1	*MELO3C005604*	geraniol 8-hydroxylase-like
2	*MELO3C005605*	subtilisin-like protease SBT1.7
3	*MELO3C005607*	geraniol 8-hydroxylase-like
4	*MELO3C005608*	nudix hydrolase 14, chloroplastic
5	*MELO3C005609*	Subtilisin-like protease
6	*MELO3C034003*	bHLH transcription factor
7	*MELO3C005610*	bHLH transcription factor
8	*MELO3C005611*	bHLH transcription factor
9	*MELO3C005612*	bHLH transcription factor
10	*MELO3C005613*	Expansin
11	*MELO3C005614*	protein of unknown function (DUF1191)
12	*MELO3C005615*	protein FIZZY-RELATED 2
13	*MELO3C005616*	protein STAY-GREEN, chloroplastic-like
14	*MELO3C005617*	agamous-like MADS-box protein AGL19

## Data Availability

The raw data presented in this study are deposited in the Genome Sequence Archive (GSA) at the National Genomics Data Center, China National Center for Bioinformation (CNCB-NGDC), under accession numbers PRJCA076428 and PRJCA076511.

## Supplementary Materials

The following supporting information can be downloaded at https://www.mdpi.com/article/10.3390/ijms27209141/s1.
