# Supplementary material for: Integrated Genetic Mapping, Transcriptomic, and Small RNA Analysis of Fruit Bitterness in Melon
Source: Int J Mol Sci. 2026 Oct 15;27(20):9141. doi: 10.3390/ijms27209141 (PMC13705163; doi:10.3390/ijms27209141)
Supplement: Supplementary file 1 [file ijms-27-09141-s001.zip › ijms-4615022-supplementary captions.pdf]

Figure S1: Sequence variation and transcript abundance analyses of four candidate bHLH genes located in the *CmBt* region. (A-D) Snapshots of the genomic regions of *MELO3C034003* (A), *MELO3C005610* (B), *MELO3C005611* (C), and *MELO3C005612* (D) visualized by the Integrative Genomics Viewer v2.14.0 (IGV). The red box indicates the position of SNP1, and the blue boxes indicate the positions of transposons predicted based on FGENESH software (https://bio.tools/fgenesh).

Table S1: Summary of RNA-seq results.

Table S2: List of differentially expressed genes (DEGs) identified by RNA-seq in young fruits (3 DAP) of $B15^{NIL}$ and B15.

Table S3: Differential expression of genes in the cucurbitacin B biosynthetic pathway between $B15^{NIL}$ and B15.

Table S4: Differential expression of four bHLH genes in young fruits (3 DAP) of $B15^{NIL}$ and B15.

Table S5: Overview of small RNA sequencing from $B15^{NIL}$ and B15.

Table S6: Summary of known and predicted miRNA in this study.

Table S7: Summary of miFam-based miRNA families and their members.

Table S8: Differentially expressed miRNAs between $B15^{NIL}$ and B15.

Table S9: All target genes of miRNAs identified by degradome sequencing.

Table S10: Predicted target genes of differentially expressed miRNAs.

Table S11: Predicted miRNAs targeting eight cucurbitacin biosynthetic genes in melon.

Table S12: Determination of cucurbitacin B content.

Table S13: Primer sequences used in this study. Supplementary data: T-plots of cucurbitacin biosynthetic pathway genes.
