# Supplementary material for: Integrated Genetic Mapping, Transcriptomic, and Small RNA Analysis of Fruit Bitterness in Melon
Source: Int J Mol Sci. 2026 Oct 15;27(20):9141. doi: 10.3390/ijms27209141 (PMC13705163; doi:10.3390/ijms27209141)
Supplement: Supplementary file 1 [file ijms-27-09141-s001.zip › T-plots of cucurbitacin biosynthetic pathway genes/cme-MIR172e-p5_MELO3C023960.2.1_1119_TPlot.pdf]

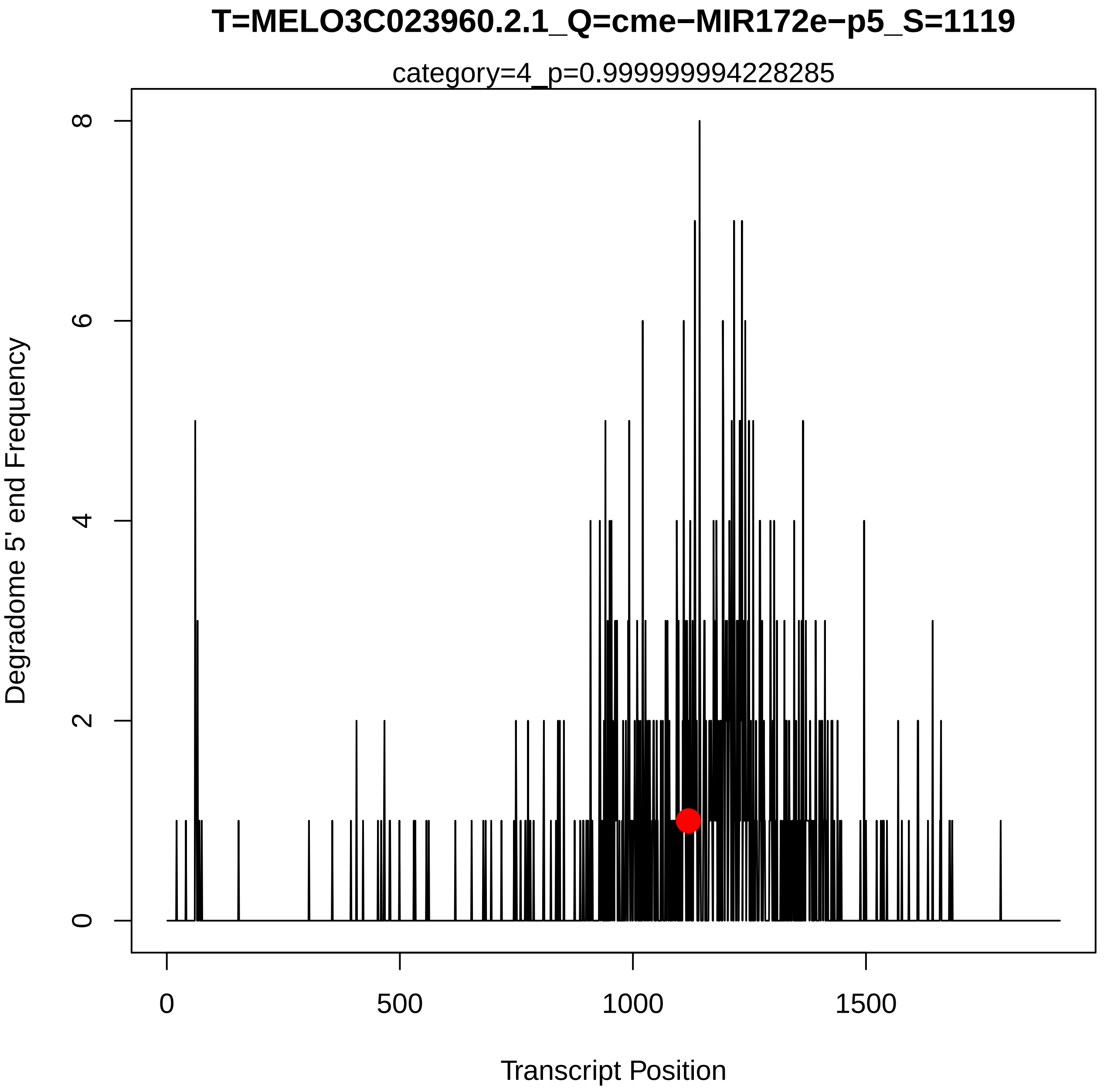
T=MELO3C023960.2.1_Q=cme−MIR172e−p5_S=1119
category=4_p=0.999999994228285
Degradome 5' end Frequency
0
2
4
6
8
0
500
1000
1500
Transcript Position
