# Supplementary material for: Integrated Genetic Mapping, Transcriptomic, and Small RNA Analysis of Fruit Bitterness in Melon
Source: Int J Mol Sci. 2026 Oct 15;27(20):9141. doi: 10.3390/ijms27209141 (PMC13705163; doi:10.3390/ijms27209141)
Supplement: Supplementary file 1 [file ijms-27-09141-s001.zip › T-plots of cucurbitacin biosynthetic pathway genes/cme-miR395a_L-1_MELO3C034003.2.1_180_TPlot.pdf]

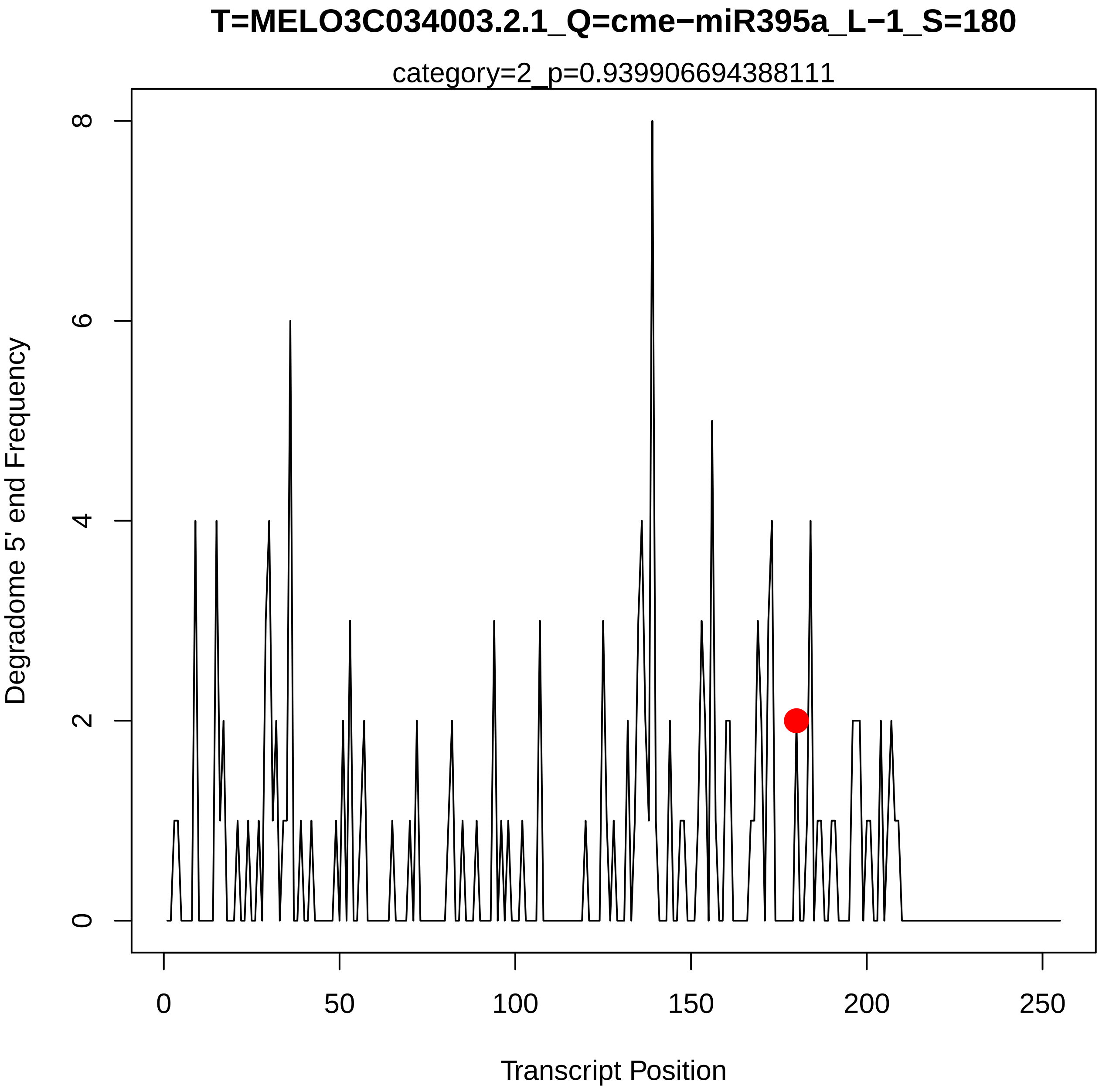
T=MELO3C034003.2.1_Q=cme−miR395a_L−1_S=180
category=2_p=0.939906694388111
Degradome 5' end Frequency
0
2
4
6
8
0
50
100
150
200
250
Transcript Position
