# Supplementary material for: Integrated Genetic Mapping, Transcriptomic, and Small RNA Analysis of Fruit Bitterness in Melon
Source: Int J Mol Sci. 2026 Oct 15;27(20):9141. doi: 10.3390/ijms27209141 (PMC13705163; doi:10.3390/ijms27209141)
Supplement: Supplementary file 1 [file ijms-27-09141-s001.zip › T-plots of cucurbitacin biosynthetic pathway genes/hbr-MIR6173-p3_1ss19AG_MELO3C022377.2.1_818_TPlot.pdf]

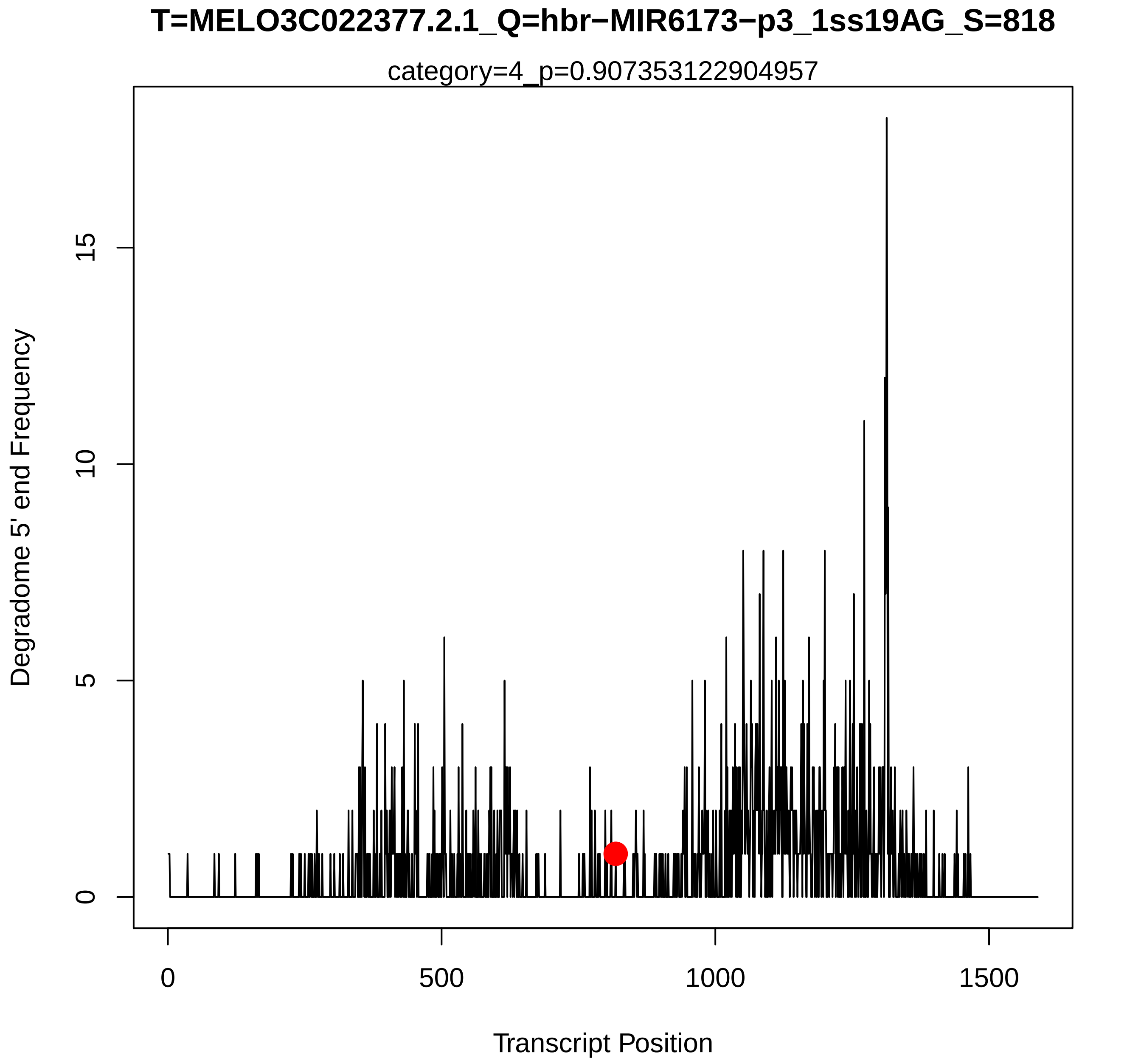
T=MELO3C022377.2.1_Q=hbr−MIR6173−p3_1ss19AG_S=818
category=4_p=0.907353122904957
Degradome 5' end Frequency
0
5
10
15
0
500
1000
1500
Transcript Position
