# Supplementary material for: Integrated Genetic Mapping, Transcriptomic, and Small RNA Analysis of Fruit Bitterness in Melon
Source: Int J Mol Sci. 2026 Oct 15;27(20):9141. doi: 10.3390/ijms27209141 (PMC13705163; doi:10.3390/ijms27209141)
Supplement: Supplementary file 1 [file ijms-27-09141-s001.zip › T-plots of cucurbitacin biosynthetic pathway genes/htu-MIR156a-p3_2ss15TG17CG_MELO3C022376.2.1_1043_TPlot.pdf]

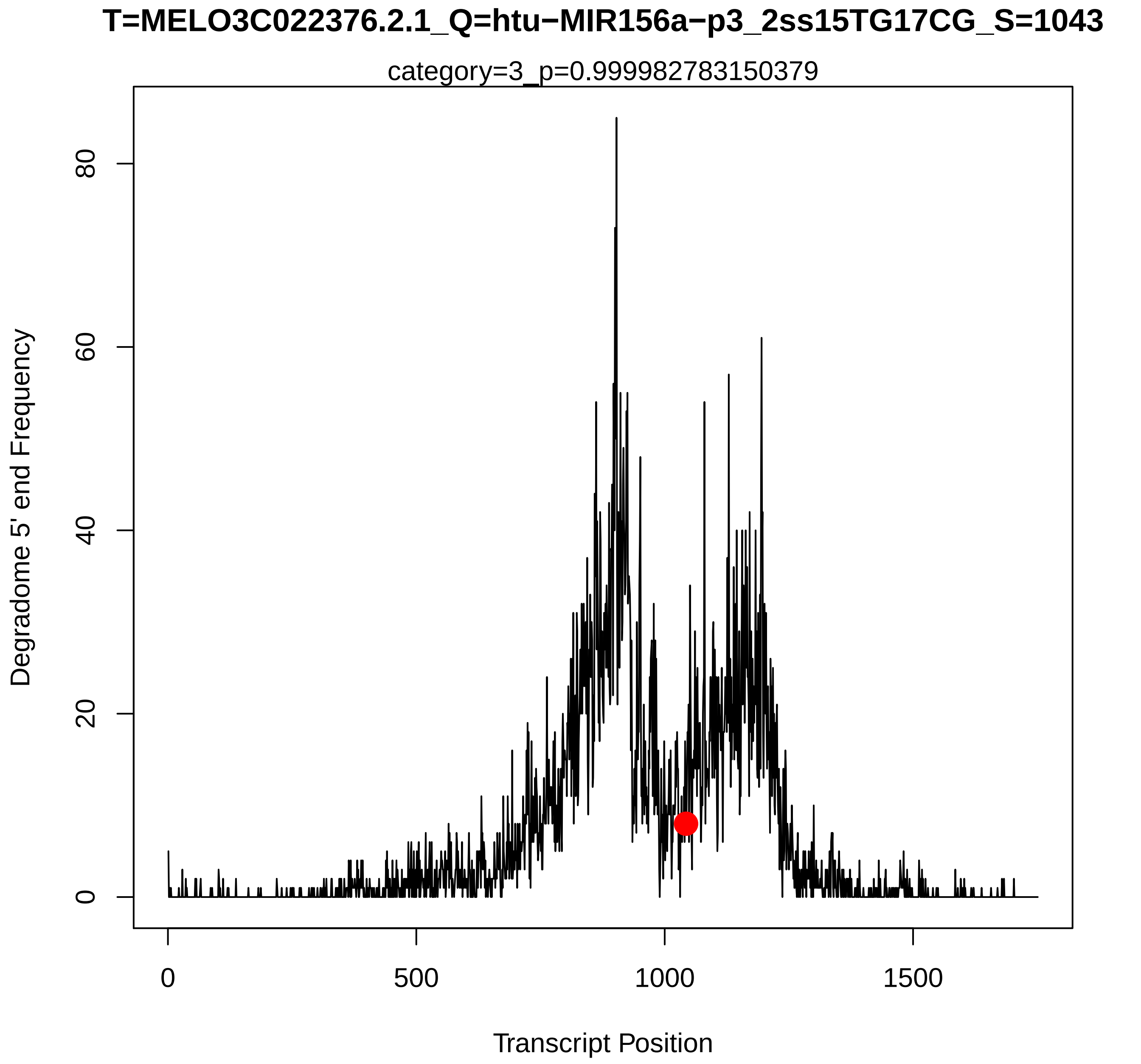
T=MELO3C022376.2.1_Q=htu−MIR156a−p3_2ss15TG17CG_S=1043
category=3_p=0.999982783150379
Degradome 5' end Frequency
0
20
40
60
80
0
500
1000
1500
Transcript Position
