# Supplementary material for: Integrated Genetic Mapping, Transcriptomic, and Small RNA Analysis of Fruit Bitterness in Melon
Source: Int J Mol Sci. 2026 Oct 15;27(20):9141. doi: 10.3390/ijms27209141 (PMC13705163; doi:10.3390/ijms27209141)
Supplement: Supplementary file 1 [file ijms-27-09141-s001.zip › T-plots of cucurbitacin biosynthetic pathway genes/lja-miR11088b-5p_L+2_2ss22CA24TA_MELO3C002190.2.1_1358_TPlot.pdf]

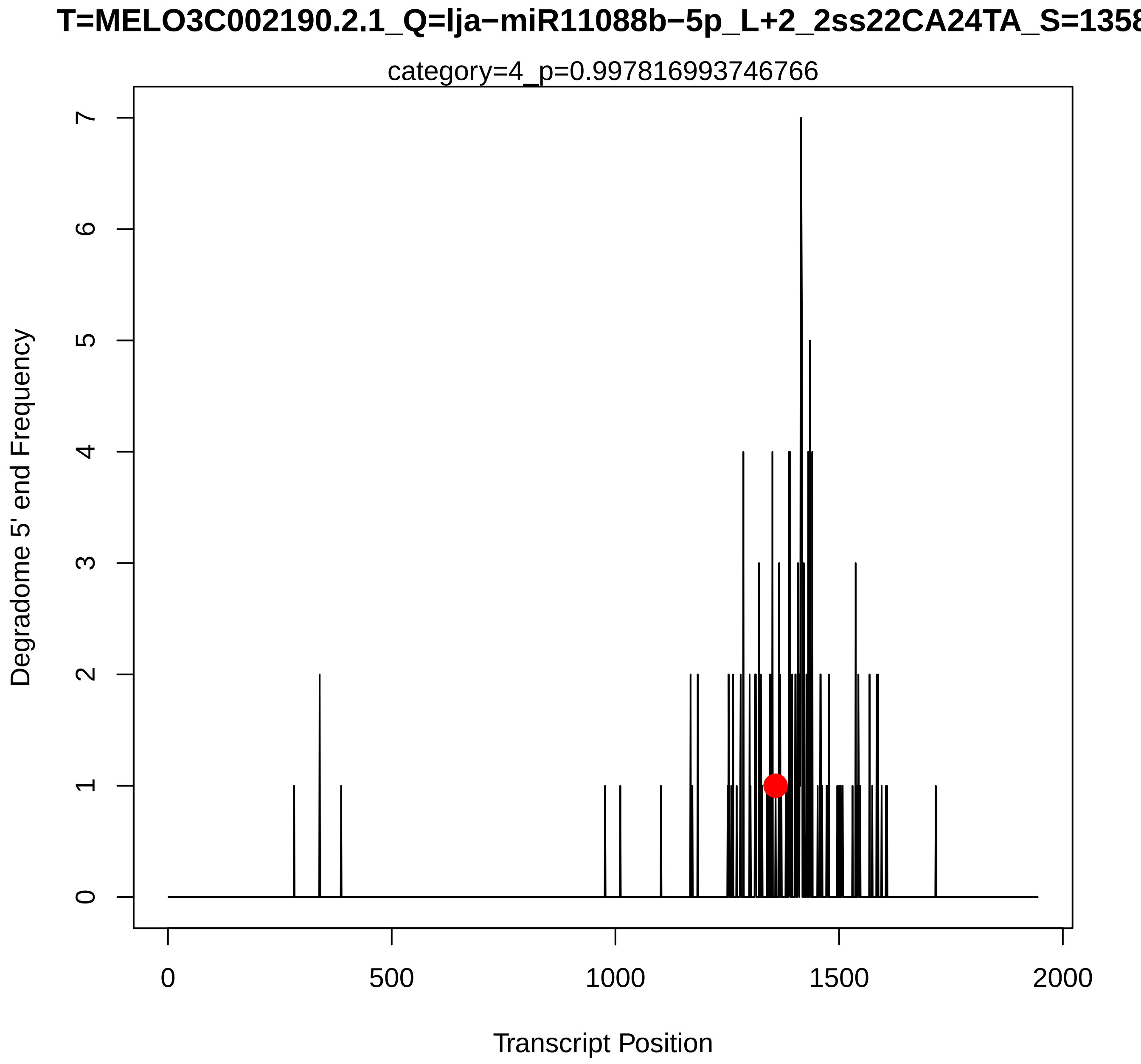

T=MELO3C002190.2.1_Q=lja−miR11088b−5p_L+2_2ss22CA24TA_S=1358
category=4_p=0.997816993746766
Degradome 5' end Frequency
0
1
2
3
4
5
6
7
0
500
1000
1500
2000
Transcript Position
