# Supplementary material for: Integrated Genetic Mapping, Transcriptomic, and Small RNA Analysis of Fruit Bitterness in Melon
Source: Int J Mol Sci. 2026 Oct 15;27(20):9141. doi: 10.3390/ijms27209141 (PMC13705163; doi:10.3390/ijms27209141)
Supplement: Supplementary file 1 [file ijms-27-09141-s001.zip › T-plots of cucurbitacin biosynthetic pathway genes/PC-3p-119091_63_MELO3C022373.2.1_1080_TPlot.pdf]

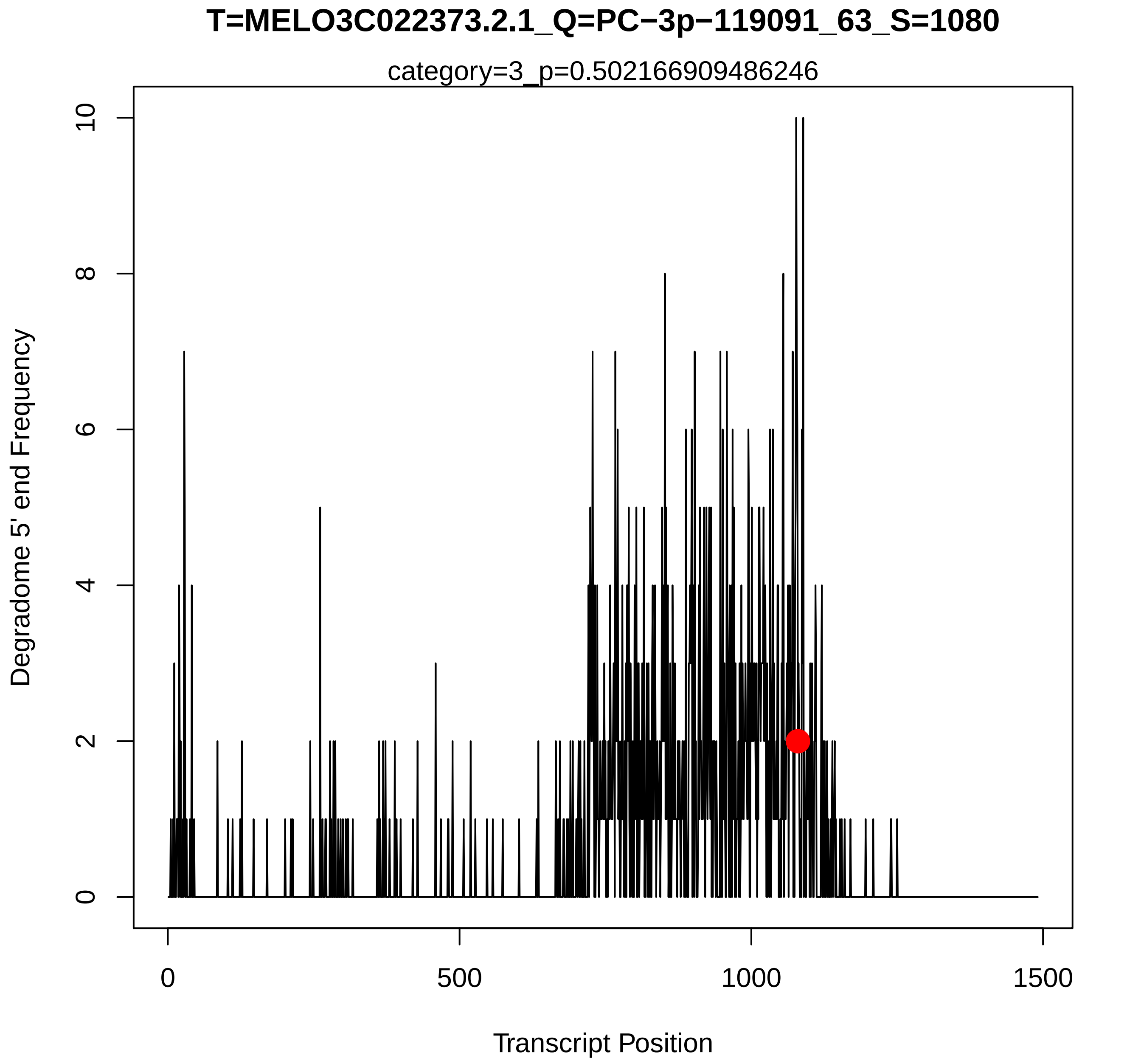

T=MELO3C022373.2.1_Q=PC−3p−119091_63_S=1080
category=3_p=0.502166909486246
Degradome 5' end Frequency
0
2
4
6
8
10
0
500
1000
1500
Transcript Position
