# Supplementary material for: Integrated Genetic Mapping, Transcriptomic, and Small RNA Analysis of Fruit Bitterness in Melon
Source: Int J Mol Sci. 2026 Oct 15;27(20):9141. doi: 10.3390/ijms27209141 (PMC13705163; doi:10.3390/ijms27209141)
Supplement: Supplementary file 1 [file ijms-27-09141-s001.zip › T-plots of cucurbitacin biosynthetic pathway genes/PC-5p-244256_26_MELO3C022375.2.1_756_TPlot.pdf]

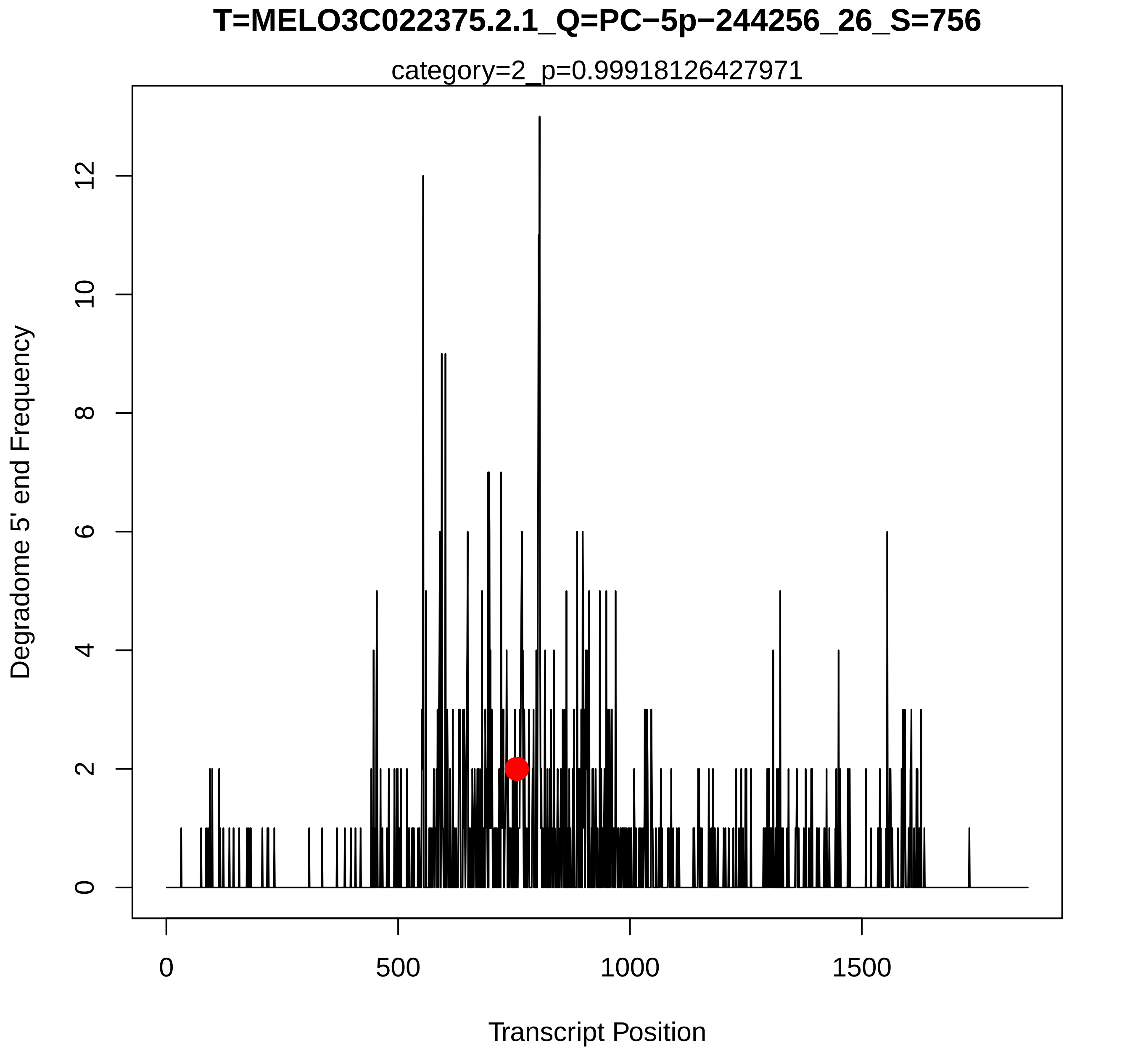

T=MELO3C022375.2.1_Q=PC−5p−244256_26_S=756
category=2_p=0.99918126427971
Degradome 5' end Frequency
0
2
4
6
8
10
12
0
500
1000
1500
Transcript Position
