# Supplementary material for: Integrated Genetic Mapping, Transcriptomic, and Small RNA Analysis of Fruit Bitterness in Melon
Source: Int J Mol Sci. 2026 Oct 15;27(20):9141. doi: 10.3390/ijms27209141 (PMC13705163; doi:10.3390/ijms27209141)
Supplement: Supplementary file 1 [file ijms-27-09141-s001.zip › T-plots of cucurbitacin biosynthetic pathway genes/PC-5p-261162_24_MELO3C023960.2.1_1248_TPlot.pdf]

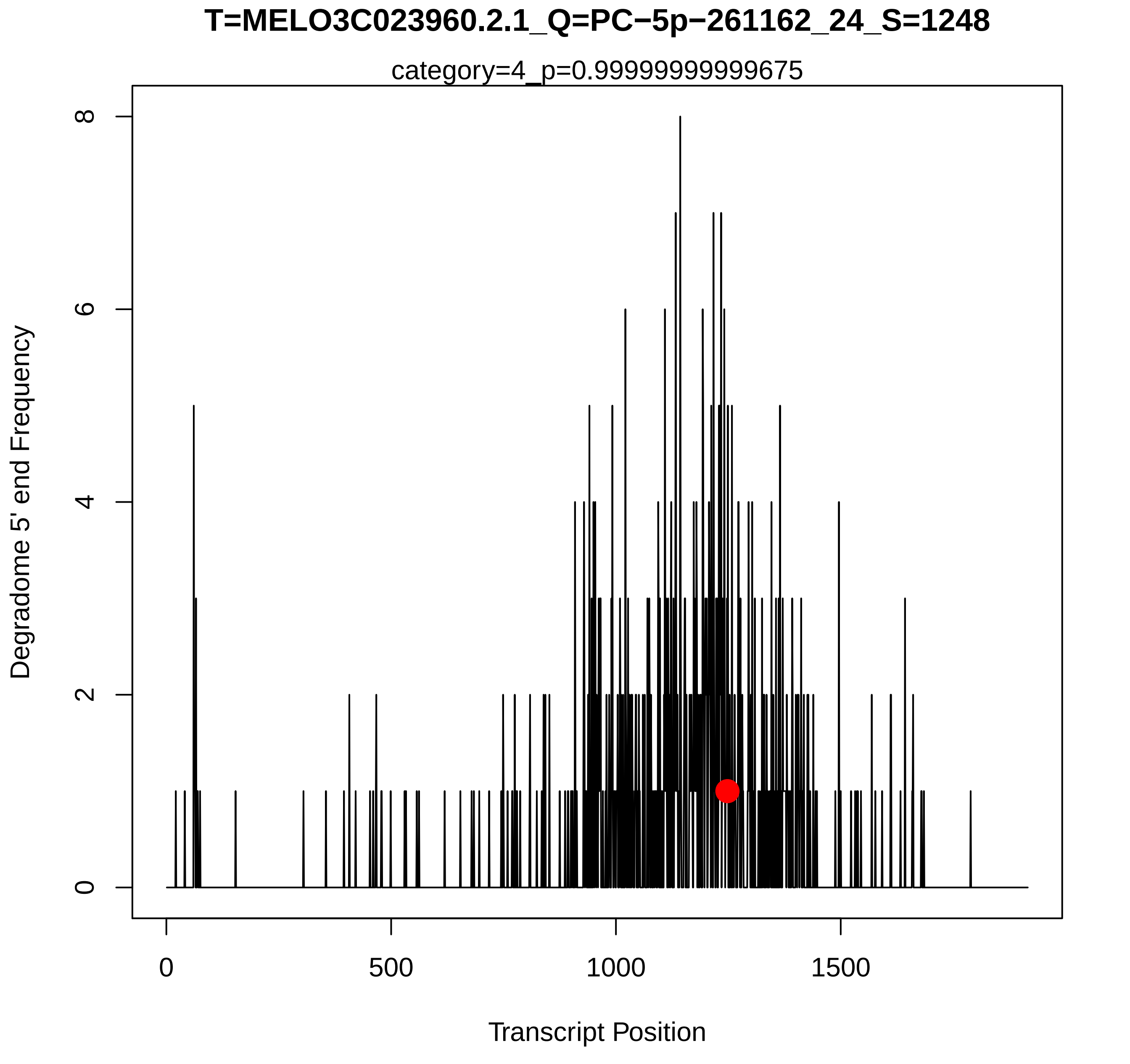
T=MELO3C023960.2.1_Q=PC−5p−261162_24_S=1248
category=4_p=0.99999999999675
Degradome 5' end Frequency
0
2
4
6
8
0
500
1000
1500
Transcript Position
